# Regeneration of Post-Agricultural Brownfield for Social Care Needs in Rural Community: Is There Any Transferable Experience?

**DOI:** 10.3390/ijerph19010240

**Published:** 2021-12-26

**Authors:** Petr Klusáček, Klára Charvátová, Josef Navrátil, Tomáš Krejčí, Stanislav Martinát

**Affiliations:** 1 Department of Environmental Geography, Institute of Geonics of the Czech Academy of Sciences, Drobného 28, CZ-60200 Brno, Czech Republic; petr.klusacek@ugn.cas.cz (P.K.); Josef.Navratil@ugn.cas.cz (J.N.); tomas.krejci@ugn.cas.cz (T.K.); 2 Faculty of Regional Development and International Studies, Mendel University in Brno, Třída Generála Píky 2005/7, CZ-61300 Brno, Czech Republic; Klara-Ch@email.cz

**Keywords:** regeneration, post-agricultural brownfield, rural development, post-socialism, public–private partnership, social care, Czech Republic

## Abstract

In the 21st century, rural communities face many challenges, including implications of dynamic population aging, a lack of social care services, and the occurrence of abandoned post-agricultural brownfields. This paper is methodologically based on the findings derived from a set of qualitative in-depth interviews with the key rural stakeholders, explores the decisive factors and limits, accelerators, and barriers governing successful regeneration of the post-agricultural brownfield in the post-socialist environment. We are using the case of the regeneration project of a large-scale former communist agricultural cooperative, located in Vranovice, the Czech Republic, to illuminate how complex and challenging the redevelopment of a post-agricultural brownfield into a social care facility for elderly people is. A wide agreement among the experts in the field of community development exists that this regeneration project can serve as a model example for other rural municipalities that are sharing similar local development issues. Our findings illustrate how important and challenging at the same time are the matters of good governance, the active and long-term participation of stakeholders in the regeneration project, and the real-life introduction of the public–private partnership concept, particularly in immensely transforming the post-socialist countryside.

## 1. Introduction

Many rural municipalities across Europe share similar issues and challenges on the global–local axis, starting with energy transition [1], digitalization [2], and adaptation to climate change on one side, and including dynamic population aging [3], depopulation of rural peripheries [4] (Sikorski et al., 2020), decay of rural settlements and underinvestment of infrastructural structures [5], a rise of rural populism [6], and the occurrence of various types of abandoned or underused sites, so-called brownfields [7] on the opposite one. A clear disadvantage of rural municipalities in finding reasonable, affordable, and effective solutions to tackle these complex challenges lies in the issue that their self-governments cannot rely on such professional and well-informed structures of knowledge providers as is the case of large cities [8]. This shortcoming is clearly evident in the field of brownfield regeneration [9]—while large cities are setting up highly skilled departments and teams of experts that deal exclusively with brownfield regeneration [10], rural municipalities are more frequently dependent on the involvement of external actors [11] and experts who are scarce in the countryside. It is quite rare that rural communities can solely depend on the local (internal) neighborhood enthusiasm or can sufficiently utilize the benefits of highly specialized EU web-based tools to support decision-making and involve stakeholders in brownfield regeneration (e.g., [12,13,14]). Consequently, following brain drain away from the rural, communities frequently lack the right set of expertise to support decision-making, which together with rapid technological and digital advancements in cities further widens the urban–rural gap [15]. 

When regenerating brownfields situated within their limits, rural municipalities often struggle with another disadvantage. Rural brownfields are frequently not as attractive for private investors as brownfields in city centers [16] or those located along regional development axes and in the proximity of economic cores [17]. The usual lack of interest among private investors then results that many (especially peripheral) rural municipalities are dependent on obtaining external public funding when regenerating brownfields. Undoubtedly, a portfolio of individual regeneration options is narrowed then as well as possible links to the needs and benefits for host communities [18]. 

We already know from the literature that among the most important factors or accelerators supporting brownfield regeneration are well-developed transport infrastructure, proximity to (or the location within) metropolitan areas, and occurrence of environmental burdens that increase the urgency for priority regeneration [19,20]. Based on the experience from our previous research (please see [10]), we can add that a clear and transparent ownership structure of brownfield sites has proven to be among the main prerequisites for advancing with brownfield regenerations. For the post-communist environment of Central Europe, some studies [16] highlight that communities with a higher development potential located in core areas have usually better chances for brownfield regeneration than rural municipalities in peripheral or semi-peripheral parts of regions. On the other hand, other studies [21] identified the original use of brownfields as an additional decisive factor governing brownfield regeneration. 

We also know that different types and groups of stakeholders tend to emphasize very different types of factors leading to brownfield regeneration. Ref. [22] for example identified that while public administrations highlight more the importance of the legislation, state incentives, and general localization of sites, investors and developers are more concerned about local-level factors (as landscape protection limits, place marketing, and previous use of the brownfield). However, it is obvious that a high-quality, well-thought-out, research-informed, and broadly set framework for the support of brownfield regeneration by national authorities is frequently considered as one of the essential factors contributing to greater efforts by local actors to revitalize rural brownfields [23].

An inspiration by examples of successful brownfield regeneration projects as catalysts for advancement with challenging projects proved to be enormously important. Good practice case studies analyzing the process, decision-making, and effects of the regeneration in an urban environment are relatively common (e.g., [24,25]), while successful cases of occurring in rural environments have been so far only mentioned as unique examples [7,26,27,28,29,30]. Moreover, the focus of these studies is rather on the final product of regeneration and its impact on neighborhoods than on the brownfield regeneration process. This has been analyzed rather rarely so far. Additionally, attention was more targeted on a large-scale rural post-mining brownfield [31] or on the sites where the occurrence of post-military brownfields and heavy soil contamination prevails [32,33]. 

This paper uniquely deals with the process of successful brownfield regeneration under specific conditions of post-socialist rural municipalities. The main objective is to identify the main factors and limits, accelerators, and barriers governing the successful regeneration of post-agricultural brownfields that match the needs of a particular community. Specifically, our case study follows the story of the regeneration of large-scale abandoned and neglected farms that negatively affected the image of the community to a social care facility. We employed qualitative research methods and techniques to reveal factors, limits, accelerators, barriers, and transferability of the knowledge gained through the regeneration process.

In the following sections, we further investigate the specificities of distribution of post-agricultural brownfields and their re-use and the significance of local actors and their empowerment in brownfield regeneration. We are particularly keen on learning more about the needs of rural communities and the activation of a variety of investors through the public–private partnership. 

### 1.1. Post-Agricultural Brownfields in Rural Space

Concerning the distribution of regenerated post-agricultural brownfields, ref. [34] ascertained that in rural regions of the Czech Republic post-agricultural brownfields have been mostly regenerated for housing, but it is clearly visible that in peripheral rural locations regenerations for farming are more frequent. This finding is in line with the effects of the ongoing urban housing crisis [35] that affects the willingness of the population to move to the rural [36]. We know that municipalities tend to actively influence the re-use of brownfields in their territories [10] to ensure that the local needs are covered. This is basically happening either indirectly through the zoning in the spatial development plans that regulate the visions of private or public investors about the future use of land (e.g., [25]) or directly as public owners of abandoned and dilapidated brownfields are under increased public pressure to regenerate (e.g., [37]). Undoubtedly, the regeneration of post-agricultural brownfields is also affecting the appearance and image of rural municipalities both externally and internally [38]. Ref. [26] claim in their study that the image of villages with regenerated brownfields positively affects tourism and leisure-time activities [39]; this is also the case for housing re-use and attractiveness for the incoming population [7]. We need to highlight here that the cooperation of various stakeholders is extremely important for successful brownfield regeneration. As [37] stress in their study, three moments must be followed when regeneration is planned with an endeavor to increase a chance for success: (i) defining brownfield problem, (ii) mobilizing networks, and (iii) leading by example, which together have a potential to define an entrepreneurial path for particular brownfield sites. Along this brownfield path, actors have a possibility to gradually evolve from passivity toward active entrepreneurship. On the other hand, as [37] warns, stagnation or even regression are also possible. As brownfield regenerations for social care needs are still rare in a post-socialist context, it is difficult to theorize, however, it is expected that dynamic aging of rural communities will feed demand for these facilities in the near future [40].

### 1.2. Local Actors, Local Activity, and the Public–Private Partnership Principle

Ref. [41] thoroughly analyzed the negotiation issues in forming a public–private partnership focused on the brownfield redevelopment. Concerning the financing issues related to brownfield regeneration, ref. [42] emphasize that even in economies with uninterrupted market relations brownfield redevelopment is increasingly driven by the availability of development grants and subsidies. This is especially relevant in rural peripheries that are distant from urban cores as engines of power and economy [43]. Specifically, for a post-socialist context, ref. [44] claim in their study that public sectors in Central Europe vastly influence brownfield regenerations by means of the subvention programs funded from the EU, national, regional, and also local level. Other financial instruments (i.e., revolving funds, guarantees, credits with supported interests, etc.) are also utilized and are highly prospective but their full potential still stays behind the expectations [45] and rather conservative nature of rural administrations. As was already mentioned above, small rural municipalities are facing limitations concerning their development possibilities due to the limited human and social capital at their disposal [46]. This shortfall is reflected in the reduced amount of time or energy that can be devoted by municipal leaders to systematically reveal the municipality’s external funding possibilities. In such cases, the development of the municipality heavily depends on external financial sources and assistance or, on the contrary, on the activation of internal potential [47]. Among the most suitable development funding possibilities in such cases is a public–private cooperation, which has been institutionalized in the form of the so-called public–private partnership (PPP) [48]. The conditions for success in the case of PPPs are highly diverse and strongly depend on local factors and specificities. Nevertheless, if we look at the successful PPP initiatives, we can say that its benefits are clear. These include access to private finance for regeneration projects, sharing the risk, usage of skills and possibilities of the private sector, and increased procedural efficiency [49]. However, PPP projects are surely not a self-sustaining solution, and this form of cooperation naturally has its drawbacks that include a long-term nature of obligations of the public sector, an impact on fiscal indicators of municipalities [50]. Some studies are even mentioning the too-complicated preparatory phase and future unpredictability of PPP projects [51]. On the other hand, having in mind possible pros and cons, a PPP path seems to belong to the ways how to progress with brownfield regeneration in the countryside and match reuses to the needs of communities at the same time [52].

## 2. Case Study, Materials, and Methods

### 2.1. Case Study Description

The case study of our interest is situated in a former socialist agricultural cooperative in the Vranovice community in the eastern part of the Czech Republic. The population of Vranovice was 2.455 as of January 1, 2021 [53]. The community was selected for in-depth study as the case study was presented in 2019 as an example of good practice of brownfield regeneration [54] at the seminar organized by the Regional Development Agency of South Moravia for brownfield stakeholders both from the public and private sectors. The specialized dissemination brochures [55,56] characterizing the site were distributed at the event, indicating that actors involved in the regeneration of the site are willing to share their knowledge gained during the regeneration process. The selected studied site is located in the South Moravian Region about thirty kilometers south of Brno, the second-largest city in the Czech Republic. Vranovice is a rural municipality surrounded by lowland agricultural landscapes where the extent of farming has been reduced due to the post-socialist agricultural transition since 1990. The total municipal area of 1.383 hectares is two-thirds covered by agricultural land [57]. Vranovice lies at the outer border of the Brno metropolitan area. The location within the metropolitan area significantly increases the population growth (by one-fifth in the last decade) as well as the development potential and financial possibilities of the municipality. 

A long-term development in the case study locality (a former large-scale socialist agricultural cooperative in Vranovice) was heavily influenced by the key historical and political milestones occurring in the Czech Republic in the 20th century. In the first half of the century, we would find solely small-scale private family farms in Vranovice where cattle, pigs, and horses were bred and small-scale crop agriculture practiced. In the 1950s, as a result of the socialist collectivization, these agricultural properties forcibly became part of a socialist agricultural cooperative. During the era of socialist agriculture (1950–1980), cattle were still bred in the area, but later, animal breeding began to sharply decline, and finally, it was abolished as it was decided to focus solely on large-scale crop production. 

During these days, the agricultural premise was extended to enable the concentration of agricultural activities into one large farm that was supposed to be an economically more efficient way of farming during the socialist era. After the end of animal breeding on the site, the cowsheds were demolished and the workshops in more expanded. 

After the Velvet Revolution and the re-introduction of the market relations after 1989, the agricultural premise was returned to the original owners as part of the restitution process. Consequently, the area of the former socialist agricultural cooperative farms was divided among several private owners, but none of them continued with practicing agriculture, resulting in the abandonment of farm buildings. 

**Figure 1:**
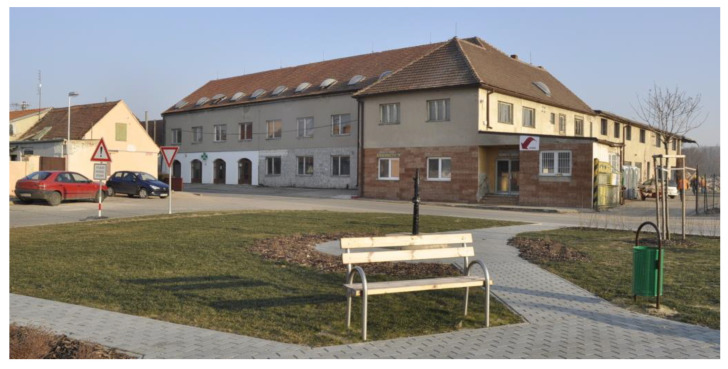
A view of the administrative building of the former socialist agricultural cooperative in Vranovice. Source: [58].

**Figure 2:**
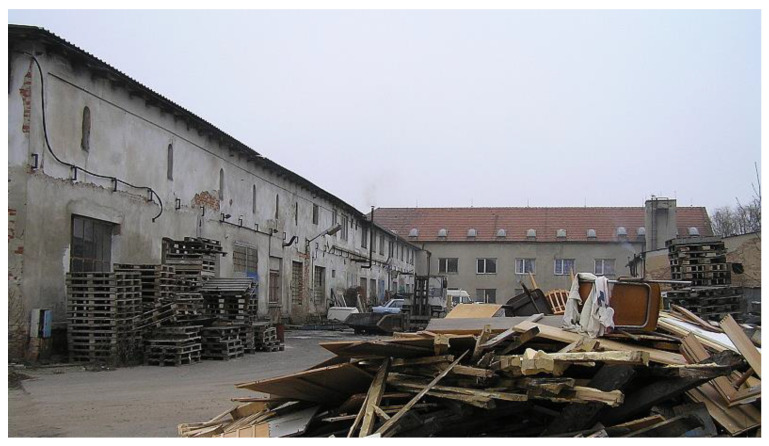
A view of the production part of the former socialist agricultural cooperative farm in Vranovice at the start of the regeneration process. Source: [59].

The former socialist agricultural farm in Vranovice has quickly started to fall into disrepair since 1991, although some farm buildings were still partially used for small-scale nonagricultural activities. At the time of the farm abandonment and decay, the whole site was not secured against the entry; only the offices and workshop buildings were locked, but the site as such was freely accessible. This resulted in frequent vandalism, theft of equipment, and the creation of illegal dumps. To illustrate how radical the decay of the farm was, the waste dumps had to be removed from time to time to avoid soil contamination and odor leakages to the community. The area of the abandoned socialist collective farms (see Figure 1 and Figure 2) created enormous problems for the community as it was situated right in the center of the settled part. Negative impacts were obvious those days as the farm was heavily dilapidated and the desolate appearance of the site degraded the environment, aesthetics, image of the community, and wellbeing of the local population.

**Figure 3:**
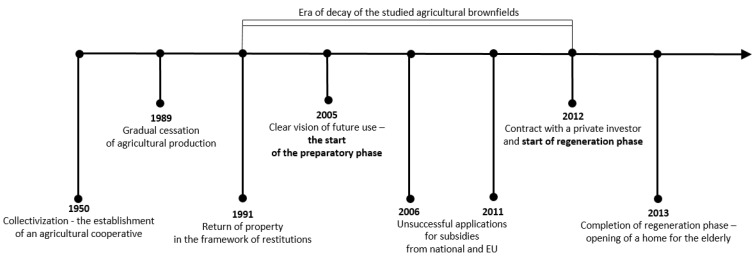
Timeline of the key milestones of the development of the regeneration project in Vranovice. Source: Authors’ own processing.

**Figure 4:**
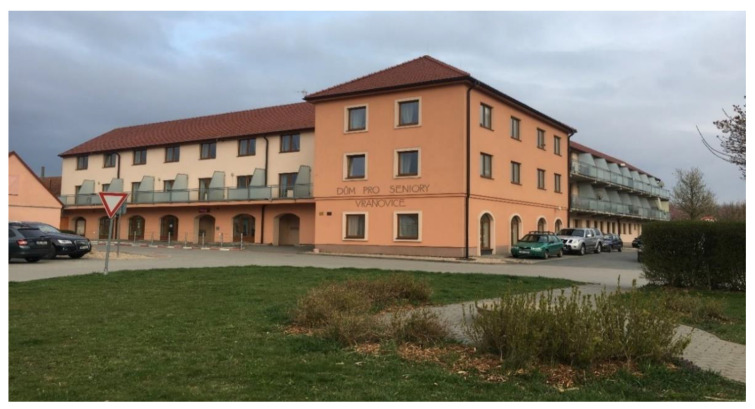
Social care home for elderly people, built on the site of a former socialist agricultural cooperative in Vranovice. Source: K. Charvátová (2021).

**Figure 5:**
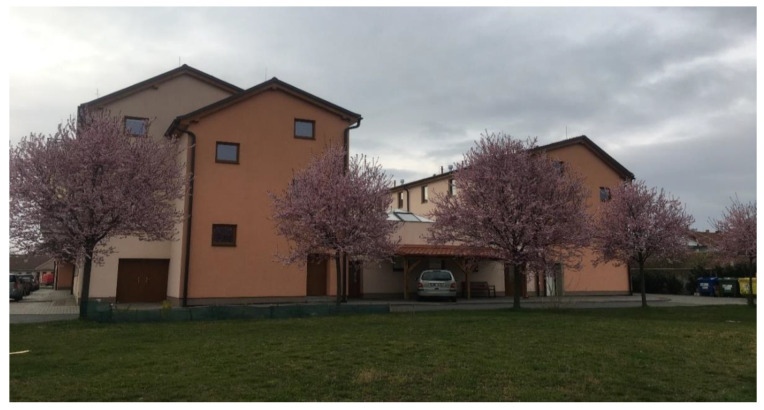
A view of the social care home for elderly people from the courtyard in Vranovice. Source: K. Charvátová (2021).

**Figure 6:**
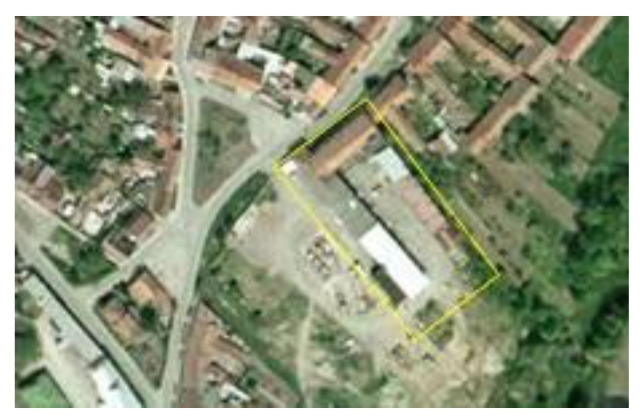
Aerial views of the site of a former agricultural cooperative in Vranovice before regeneration in 2003. Source: [60].

**Figure 7:**
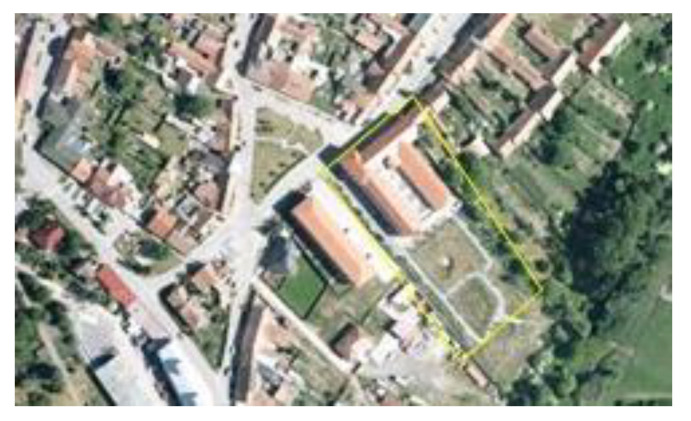
Aerial view of a new social care home for elderly people with a new park in 2015. Source: [61].

At the beginning of the 21st century, growing negative phenomena associated with the occurrence of abandoned post-agricultural brownfields in the center of the municipality began to gradually motivate local authorities in Vranovice to seek regeneration possibilities. In 2005, the first important step was conducted and the abandoned agricultural farm was purchased. The vision of the mayor was a social care home for the elderly people that would be eventually built here in combination with other multifunctional uses (like catering facilities for local elementary schools, and other public, dance, and social halls for the community, hairdresser, and pharmacy). A large-scale regeneration project naturally required enormous investment inadequate to the scale of the budget of a small municipality. Therefore, the representatives of the municipality decided to apply for external funding. However, after several unsuccessful attempts to get the project funded, attention was turned to the funding by means of the public–private partnership. The extension of time, when funding for the regeneration was sought, was finally shown to be indeed long, as took seven years. Finally, after many years of the preparatory phase, the following construction activities took another two years (see Figure 3 for the timeline). Thanks to this public–private partnership concept, the regeneration project (please see Figure 4 and Figure 5 for the final regeneration outcome) was completed in 2013 and since then the site has been successfully used both for elderly people and for a wider local public. As a part of the regeneration, a significant change in the usage of land within the site has been achieved, which can be nicely illustrated with aerial photographs from times before and after the regeneration (please see Figure 6 and Figure 7). The basic characteristics of the regeneration project and a systematic overview are listed in Table 1.

### 2.2. Methods and Data

Our methodology is based on analyses of the information provided by ten interviewees, which were conducted from February to May 2020. The interviewed communication partners were carefully selected to represent diverse actors involved in this regeneration project. Both private and public sector stakeholders, as well as local inhabitants, were interviewed. We conducted the interviews with two groups of actors, both internal and external (please see Table 2 for the list of interviewees). We carried out five interviews with internal actors who were directly involved in the redevelopment of the site. During these interviews, attention was principally focused on the preparatory and implementation phase of the regeneration project and on the ex-post evaluation of the final result. Another four interviews were conducted with external actors who were asked to critically evaluate the final regeneration. One of the interviewed actors (a person living near the site) was also asked about the public discussions organized by the municipality in the preparatory phase and on the impact of construction work on the environment in the implementation phase.

Table 2 shows the complete list of interviewees after the topic saturation was reached. The list of communication partners to be interviewed was assembled by an in-depth study of the materials and information about the regeneration project (local newspapers, minutes from public hearings, project documentation, etc.). To ensure that all important perspectives are covered, diverse representatives of various groups of stakeholders were approached. All the interviews were conducted with the physical participation of both parties and lasted for circa 90 min. The interviewees gave their consent and were thoroughly informed about the aims of our research and how the information provided will be handled. Interviews were recorded, which was communicated and agreed upon beforehand interviews. The oral records from interviews were subsequently rewritten and the transcripts were coded and analyzed using the Atlas.ti software. We used the coding system in the Atlas.ti software and identified the main accelerators and the main barriers related to the regeneration process of the studied site. To avoid any breach of anonymity, the personal information of the interviewees was stored separately. Storage of all materials was offline for security reasons.

## 3. Results. Regeneration Journey: How a Small Community Can Achieve Big Things

### 3.1. Vision of the Project and the Search for Funding

The dilapidated area of a former agricultural cooperative located in the municipal center of Vranovice was often discussed as an issue during the meetings of the municipal council in the first years of the 21st century when pressure from the local public gradually grew. A member of the municipal council described these initial discussions: “In the debates it was frequently said that we simply have an old building in our municipality that is dilapidated and unused, but these are not things we would normally solve; we just knew that something has to be done…it was a matter of time…it was unaesthetic and, of course, it was a pity for the building.” The mayor came up with the vision of regeneration later, in 2005. The initial motivation for dealing with the site was obvious (as is stated above), but the municipality’s priority was to rebuild the former area of the agricultural cooperative into a social care home for elderly people as the issue of dynamic aging is urgent in the community. The mayor commented on this approach: “We thought for a long time about everything and thanks to the fact that it was in the center of the village and that the age of people in the community was increasing…So in this spirit we were looking for what the village lacks the most, and somehow we tried to satisfy the need for the social care home for elderly people or to do something for our senior inhabitants and at the same time get rid of the neglected building that was not in municipal ownership.” A clear vision of what to do for local people and how to address dynamic aging as a major issue in the community seemed to be the driving force behind the whole regeneration project. Strong support from the local community for the delivery of the project can also be highlighted as a decisive accelerator. 

The problem soon arose as a former agricultural cooperative farm was not owned by the municipality. The municipality took advantage of the plan of the private owner who planned to build an apartment building on the site, but it was too large for this purpose. So, the municipality exchanged the site with the owner for another property (abandoned health care center). As was stressed by the interviewees, it was lucky that just a symbolic financial compensation was requested for the exchange of properties. The architect commented on the motivations for the exchange: “The mayor actually wanted to use a former health center in some way, and then private owner of a former cooperative farm, who actually had a construction business, wanted to regenerate the farm. But the health center site was too small to be used as a home for elderly people and on the other hand, for the private owner, the area of the agricultural cooperative was too large for a new apartment building… So, the exchange was made.” The mayor of Vranovice emphasized that “the area of the former agricultural cooperative farm had a great advantage in terms of its redevelopment potential into a social care home for elderly people. There was a large plot of land available, and it was possible to create a large garden where residents could spend their afternoons and so on. They can go outside; they don’t have to be closed in the rooms all the time… ”.

As it showed later, the acquisition of a dilapidated and neglected post-agricultural brownfield was only the first and the easiest step to be done. Fundraising the finances for the regeneration proved to be a far more challenging issue than was originally expected. The planned project turned out to be one of the largest investments in municipal history (EUR 1.92 million). In the end, the process of seeking suitable funding gradually evolved into a several-year struggle. 

As the beginning of a funding search covered the period after the Czech Republic’s accession to the European Union in 2004, the expectations about quick accessibility and availability of EU funding were unreasonably high. There is no doubt that a lack of experience with getting funds for such a large project resulted in unrealistic expectations. For several years, the local government officials unsuccessfully sought funding from various subsidy programs. Moreover, it showed later that the national level subsidies were not available at that time and EU subsidies were not intended for the development of a social care home. This illustrates how a lack of fundraising experience caused the issue that could have been avoided.

The most realistic possibility to get public funding for the project showed when the regional authorities (the South Moravian Region) introduced the plan to build several social care homes for elderly people that would be financed through a loan from the European Investment Bank. The decision about the loans provided by the European Investment Bank was in the competence of the regional authority. As available funding was not able to fund all the projects, just seven other projects within the region got funding, while Vranovice stayed right below the line of funded projects in eighth place. The mayor of Vranovice characterized the situation: “We negotiated with the South Moravian Region for about a year, because the region was supposed to take out loans for the project of seven houses for the elderly in the South Moravian Region at that time… however, we finished at eighth place.”

Later, when EU and other public funds were shown not to be obtained, the representatives of the municipal government decided to follow the path of the public–private partnership project. This idea looked realistic as the municipal government managed to find a private investor with experience in operating social care services for elderly people. The first step in the plan was to sign a preliminary agreement about a future purchase, where the private partner promised to purchase the part of the site and provide social care services. At the expense of the municipality, the complete construction of a social care home was carried out and after the completion of the basic construction, a predetermined part of the whole house was sold to the private investor as agreed. 

### 3.2. Architectural, Technical, and Social Challenges Related to the Regeneration Process

From an architectural point of view, two regeneration variants were originally considered. The first option was to demolish the entire area of the former agricultural cooperative farm and after that, build a new social care home for elderly people. The second option considered partial demolitions, in which older buildings on the site would be demolished and newer ones would be rebuilt for a social care home. In the end, the not so bad state of construction and financial savings caused that the second variant to be carried out. Thus, in the end, a combination of partial reconstruction and partial demolition was realized. The deputy mayor ex-post assessed the decision: “In the end, we found that the decision was perhaps not the happiest, because during the regeneration a lot of hidden problems were discovered… for example, there was no wreath that holds the building together and is important from the point of view of statics…some walls were found to be lined with very weak hollow bricks…there were poor quality or no foundations in some parts. So we even had to use some additional micro-piles under the building to make it stronger…to be honest, if it was built completely new after a complete demolition, it might be better.” On the other hand, although the rebuilt office building of the former agricultural cooperative was not historically significant, one part of it with vaulted ceilings was left in its original design. It was an architectural element that most members of the municipal council wanted to preserve as part of the local history, even without the legal protection status. It was also necessary to deal with waste after demolitions that had to be disposed of in an ecologically friendly way. For example, in the workshops where agricultural machinery used to be repaired, the surfaces were contaminated by old oils and other liquids. The mayor noted during the interview that during the era of the decay of the area ”black illegal landfills were created here, which completely degraded the center of our rural village.” Contrarily, not everything that was discovered during the regeneration was problematic. For example, a forgotten well was discovered, which the mayor commented: “As the demolition of workshops used to repair agricultural machinery took place, a well was found there. They had it covered and her existence surprised us. Fortunately, the well was outside the building we were constructing, so we decided to keep it… nowadays it is in the garden and it is functional… ” From the point of view of possible social conflicts related to the regeneration, sufficient and transparent communication with the actors was frequently mentioned and thus found to be of crucial importance; this is especially the case for people living in the neighborhoods a regenerated site. It was usually highlighted during interviews that each individual construction activity usually negatively affected the neighborhoods with an increased level of noise and dust that burden the wellbeing. According to the deputy mayor “there was no doubt that the regeneration bothered neighbors, as every construction does, but thanks to the fact that it did not take even two years, and even those neighbors knew that they would get rid of a permanent problem there, so they knew that they had to endure the dust during construction activities.” The comment was also made that it was important that the regeneration project was thoroughly discussed not only at the meetings of the municipal council but also during public hearings, where detailed information was provided and criticism reflected. Additionally, the FAQ website has been set up on the community webpage to assist the locals with the information about the regeneration. A couple of interviewees noted that the biggest criticism surprisingly occurred when the plan to turn the surrounding gardens around the brownfield site into a park with benches. Criticism was unexpected as this part of the regeneration plan was considered to be originally the less problematic. The problem arose as gardens were previously used for small-scale farming activities, whose users opposed the change. The mayor described the change in approach: “The first reactions of the people who grew potatoes, carrots, and other crops there were negative… they did it there for 40 years, but, of course, the development of the rural community is modernizing and moving forward… it was a bit controversial, but when I talk to these people today after those years after the regeneration, they tell me I was right, it’s calm here, it’s nice here…”. A neighbor living just next to our case study area summed up the development in the locality: “I remember the origin of the collective farm, but after its demise the site looked very sad. I appreciate that the reconstruction revived not only the buildings, but also the surroundings such as sidewalks and roads or a park on the adjacent square.” 

### 3.3. Regeneration Result and Ex-Post Evaluation of the Project 

The interviewees agreed that the asset of the regeneration project includes that the final product, i.e., a social care home for elderly people serves, both directly and indirectly, the vast majority of social groups in the community and beyond. The upper floors of the regenerated building, which are owned by the private social care service provider company, provide high-quality housing in which self-sufficient elderly people and also people with disabilities live. On the ground floor of the building, which still belongs to the Vranovice municipality, a pharmacy, hairdresser, and a dining room are situated that serve not only seniors but also students of the local primary school and other members of the local public too. Additionally, several new jobs were also created at the site. Moreover, a small part of the regenerated space is used by the Mothers “Club and the Pensioners” Club that both significantly positively contribute to the social development of the community. According to the deputy mayor, the main benefit of the regenerated site is the operation of social services for the locals and spaces used for culture and catering. Specifically, he considers the dining room to be the most pleasant element “which is really modern and serves both children, citizens, and seniors. It is used by the really general public and it is quite used for various commercial celebrations, weddings, various birthdays, and smaller cultural events, there are for example wine exhibitions and the like, there was a dance courses and so simply, it is a smaller hall, which is such pleasant. By completing the regeneration, we managed to lay the foundation stone for the emerging Social Program of the Municipality of Vranovice, which is not only a program for seniors, but it is a program designed for all target groups who need help.” The project seems to be successful from a longer-term perspective, which the mayor commented: “In fact, after 6 after 7 years, I can say that it still works the same way. It’s utilized to the maximum, there aren’t even any free beds or free apartments, so it definitely works well!”

The studied regeneration case of a post-agricultural brownfield into a social care home was also appreciated by many external actors outside the municipality. For example, in 2014, the project received the award from the State Housing Development Fund and the Foundation for the Development of Building and Architecture for the best building intended for housing. The regeneration project also succeeded in a competition of sixty-four construction projects implemented throughout the Czech Republic [62]. The representative of the State Housing Development Fund described the reasons for positive evaluation: “There were two main reasons. One reason concerns technical matters, as it is a perfectly performed reconstruction of the existing building, including the revitalization of the surroundings. The second reason is that the home for the elderly is a natural part of the village, in which there is a natural intermingling of two groups—on the one hand clients and on the other public.” The representative of the Foundation for the Development of Building and Architecture also commented on the result of the regeneration project in a positive way: “This project is a dream come true for every village the size of Vranovice. How we managed to connect a historic building in the village with the intention of the village management and a private investor is a model example of a possible solution, which should be known and presented throughout the Czech Republic.”

From the point of view of the long-term success of the project, it is inevitable that the home for the elderly has enough residents to be economically profitable. A representative of the company operating a home noted that “The interest is shown by seniors from a radius of about thirty-five kilometers around Vranovice, including those interested in Brno as the center of the region. Preference is given to domestic applicants, but in the case of vacancies, these positions are filled by applicants from the surrounding rural municipalities or from Brno.” In this context, it is important that even more demanding residents who originally lived in large cities express satisfaction with the conditions in the home. This can be illustrated by the evaluation of one of the residents: “It’s unbelievable, during the construction I came here to watch the building grow. The surroundings were neglected, I couldn’t imagine what would change here… I’m excited about the whole project. You can really see a lot of work here… I originally applied for a home in a home for the elderly in Brno, when I learned from a friend about a project in Vranovice, I did not hesitate… my immediate decision to settle here certainly did not disappoint me.” 

## 4. Discussion 

**Figure 8:**
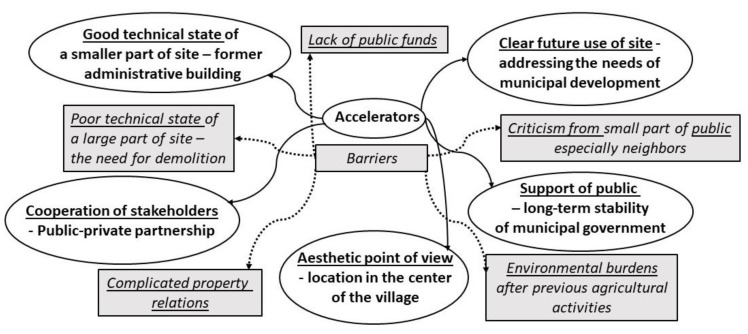
The most important accelerators and barriers governing the successful regeneration of post-agricultural brownfield in Vranovice. Source: Authors’ own processing.

Based on the rich information gathered from the interviews conducted, the main accelerators (see Table 3) and the main barriers (see Table 4) behind the regeneration of post-agricultural brownfield in Vranovice were identified. The findings are also graphically summarized in Figure 8. 

Concerning accelerators, communication partners the most frequently mentioned a long-intended intention to regenerate the site and the favorable location of the building as the most important accelerators driving the regeneration. Other accelerators that further contributed to the re-use of the dilapidated area of the former agricultural cooperative included preserved existing construction structure of the building and intense cooperation with and among individual actors. Specifically, the location of the post-agricultural brownfield and the current state of the site, in which the existing infrastructure can be used, are among the two accelerators consistent with the findings in the study by [20], who focused on the origins of post-agricultural brownfields in the Czech Republic. The communication partners also agreed that the regeneration was further stimulated by the sequence of events that occurred during the exchange of buildings with the private company within the village, as well as by positive feedback from citizens who desired to re-use abandoned sites in their neighborhoods. There is no doubt that pressure from the community towards the municipality officials sped up the regeneration helped the realization of the project. The support from the municipality leader was important too as resulted in the continuous information flow towards the groups of stakeholders with various interests. As another accelerator, an overall persistence and consistency in promoting the regeneration project can be added as it did not deter even after several unsuccessful rounds of searching for external funding.

In addition to the accelerators contributing to the regeneration of the site, the communication partners were also talking about the barriers or obstacles that negatively affected the regeneration process. The interviewees agreed that the biggest shortcoming in the initial stage of the regeneration was a poorly conducted survey of the actual technical state of the buildings, which consequently led to the increase in regeneration costs. Ref. [20] mention in their study that the opportunity for the redevelopment of post-agricultural brownfields lies primarily in gaining support from the EU and national programs, but this finding was not confirmed in our case. According to our communication partners, too much focus on searching for external funding overshadowed other internal possibilities and prolonged the regeneration process. The mayor of Vranovice interestingly summed up the lessons learned from the regeneration story: “It is important to say what the municipality needs from the brownfield, and only then to seek the funding. It must never be the other way around if someone says, public subsidies for this and that are available today, so we will have it here, no it is not possible. It has to be according to, really, what the village needs, what fits into that village.”

Other significant issues were identified in the ownership relations whose complexity tends to obstruct the regeneration process and in the fears of the population living in the neighborhoods that the regeneration will bother their wellbeing during the construction works. According to the authors of [20], abandoned post-agricultural premises incline to attract risky environmentally controversial activities resulting from the previous agricultural activities (like storage of hazardous substances, fertilizers, plant protection products, fuels), but also from the subsequent abandonment of agricultural areas (creation of black landfills for municipal waste and illegal landfills for hazardous waste). The communication partners highlighted these findings and considered the disposal of non-disposed waste and other substances as one of the barriers to regeneration. On the other hand, when talking about this particular regeneration project in Vranovice, this problem has not been detected.

## 5. Conclusions

The studied project showed that solely relying on funding from the public subsidy programs can prolong the regeneration period. This finding is in line with the findings of the previous studies from Brno (Czech Republic). While privately funded regeneration projects have already been successfully completed [25], the regeneration of publicly funded former prison buildings organized by the city of Brno is constantly postponed consequently to the expected but unsure funding [24]. The implementation period of the regeneration projects, which are managed directly by municipalities is extended then and usually covers several election periods, while the concrete regeneration projects may lose the political support of elected members of the municipality boards. Therefore, the regeneration may be postponed again or even suspended. Although this was not the case in Vranovice, where stable political support at the municipal level for regeneration was ensured, this is frequently mentioned in other studies as a significant regeneration problem (e.g., [22,63]). It truly seems that good governance is of crucial importance not only on higher hierarchical levels of governance such as the EU, national, or regional levels [10], but also, and we would say primarily, on the level of individual municipalities and specific brownfield sites. Capacity building of the rural community administrations deserves more attention and support as plenty of seemingly too complex problems to be solved on a local level might find the solution if the right skills and competencies are locally developed and advanced [64]. 

We are very much aware of the limitations of our study. In particular, our findings are primarily transferable to post-communistic countries with similar socio-cultural and political trajectories and milestones in the after-WWII period. On the other hand, the topic is relevant for a larger group of countries of the developed world as aging in rural peripheries is comparably challenging. Using the case study in Vranovice, we can see that local enthusiasm, a clear vision, and relentless drive within rural communities in promoting the brownfield regeneration project can result in a successful and followable path of endogenous local development. In this context, it might be mentioned that the Vranovice municipality can be described as a socially coherent rural community that is very much rooted in local traditions that are rather rare in other parts of the Czech countryside (more religious people with a strong tradition of folk legacy celebrations can be found here). It seems to be more difficult to jointly agree on a new development vision in communities that lack a strong sense of communality and togetherness (e.g., [33]). Future research activities in this field might focus on seeking the answer to whether centralized brownfield regeneration strategies are sufficiently taking the specifics of endogenous development of peripheral communities into account.

## Figures and Tables

**Table 1:** The selected basic characteristics of the regeneration project in Vranovice.

Characteristic	Description
Investments in the construction of the social care home for elderly people	EUR 1920 million
Investments in the park and public greenery around a new home	EUR 77 thousand
Total capacity of elderly people	80 elderly persons
Kitchen capacity—catering services for elderly people, students of local elementary schools, and other public	350 meals a day
Start and completion of the regeneration project	March 2012–May 2013

Source: Authors’ own processing.

**Table 2:** Basic characteristics of interviewed communication partners.

Communication Partner	Sex (F/M)	Education	Age Category (Years)	Position
No. 1	M	University	50–60	Mayor—Internal actor
No. 2	M	High school	40–50	Deputy Mayor—Internal actor
No. 3	F	University	30–40	Member of municipal council—Internal actor
No. 4	F	University	40–50	Architect—Internal actor
No. 5	M	University	60–70	Representative of a private company operating the home—Internal actor
No. 6	F	University	30–40	External actor—Project evaluator No. 1—Representative of the State Housing Development Fund
No. 7	M	University	60–70	External actor—Project evaluator No. 2—Representative of Foundation for the Development of Building and Architecture
No. 8	F	Basic education	70–80	External actor—Person living in the vicinity of the site
No. 9	F	Basic education	70–80	External actor—Residents of the social care home

Source: Authors’ own processing.

**Table 3:** Information about the main identified accelerators of the redevelopment process related to the case study area.

Code No.	Accelerators	Frequency	Description
1	Clear future of use of site	29	Long-term plan to create facilities for seniors, school canteen, and other services important for the municipal development
2	Aesthetic point of view—location in the center	22	Efforts to remove the ugly dilapidated building devaluing the center of the rural community
3	Good technical state	15	Preservation of the historical character and the existing construction of the administrative building to save funds intended for regeneration
4	Cooperation of stakeholders	14	Good cooperation between public and private sector actors in all phases of the redevelopment project
5	Support of public	10	Suggestions and positive responses for finding a new use from the citizens of the municipality. Long-term support in municipal elections—the same mayor was re-elected in several terms

Source: Authors’ own processing using ATLAS.ti software (Scientific Software Development GmbH, Berlin, Germany).

**Table 4:** Information about the main identified barriers of the redevelopment process related to the case study area.

Code No.	Barriers	Frequency	Description
6	Poor technical state	17	Disrupted statics of majority of buildings leading to the additional costs (demolition, ecological disposal of materials)
7	Lack of public funds	16	Time-consuming negotiations in repeated attempts to obtain public subsidy titles that have not been provided
8	Complicated property relations	12	Complicated acquisition of the building into the ownership of the municipality and a demanding search for a private company buying premises for the operation of services for the seniors
9	Criticism from public	11	Concerns of citizens living around the site about future site changes. Complaints regarding noise and dust caused by construction works
10	Environmental burdens	10	Disposal of black dumps, tires, oils, insulation, and other types of hazardous waste and animal pests

Source: Authors’ own processing using ATLAS.ti (Scientific Software Development GmbH, Berlin, Germany).

## Data Availability

The data presented in this study are available on request from the corresponding author.
